# P197: Futility of perioperative urinary analysis before elective total joint arthroplasty

**DOI:** 10.1186/2047-2994-2-S1-P197

**Published:** 2013-06-20

**Authors:** I Uckay, L Pagani, C Bouvet, A Agostinho, P Hoffmeyer, D Pittet

**Affiliations:** 1 Infection Control Programme, Geneva University Hospitals, Geneva, Switzerland; 2 Orthopaedic Surgery, Geneva University Hospitals, Geneva, Switzerland

## Introduction

The search for asymptomatic bacterial urinary tract colonization (UTC) and its eradication before elective hip and knee arthroplasty surgery is controversial, but reflects widespread practice. The influence of perioperative antibiotic prophylaxis on the dynamics of UTC is unknown.

## Objectives

We investigate the role of preoperative urinary tract colonization in patients undergoing elective joint arthroplasty.

## Methods

Prospective observational cohort study (November 2011-October 2012) with urine analyses before and 3 days after surgery. Patients with symptomatic infections or long-term urinary catheter carriage were excluded. Post-discharge surveillance included questionnaires to patients and general practitioners at 3 months.

## Results

480 asymptomatic patients (370 hip arthroplasties; 297 females; median age 71 y) were enrolled. On admission, 171 patients (35%) had bacterial UTC, mostly due to *E. coli*. Urine analysis revealed also 169 episodes of leukocyturia. Almost all (95%) received a single-dose perioperative prophylaxis of cefuroxime 1.5 g IV. Median duration of postoperative urinary catheter carriage was 0 days (range, 0-13).

On postoperative day 3, urinary analysis was abnormal in 90 episodes of leukocyturia and 198 episodes of UTC, respectively. Day 3 -bacterial UTC was different from preoperative sampling among 50% of patients and microbiological results revealed a higher proportion of Gram-positive organisms.

Only 30 patients (6%) developed a symptomatic urinary tract infection during a follow-up of 3 months; one-third of pathogens were unrelated to those found during hospitalization. All symptomatic infections were treated with oral antibiotics. There was no seeding of joint prostheses. Estimated minimal laboratory costs for preoperative urinary analyses were € 27,300.

## Conclusion

Pre- or postoperative routine urine evaluation of asymptomatic arthroplasty patients is costly and only moderately predicts the pathogen of a potential urinary tract infection. If symptomatic infection occurs, a targeted individualized antibiotic therapy prevents urosepsis and hematogenous spread to joint prosthesis.

## Disclosure of interest

None declared

